# Supplementary material for: Melanoma Mediated Disruption of Brain Endothelial Barrier Integrity Is Not Prevented by the Inhibition of Matrix Metalloproteinases and Proteases
Source: Biosensors (Basel). 2022 Aug 19;12(8):660. doi: 10.3390/bios12080660 (PMC9405625; doi:10.3390/bios12080660)
Supplement: Supplementary file 1 [file biosensors-12-00660-s001.zip › biosensors-1813649-supplementary.pdf]

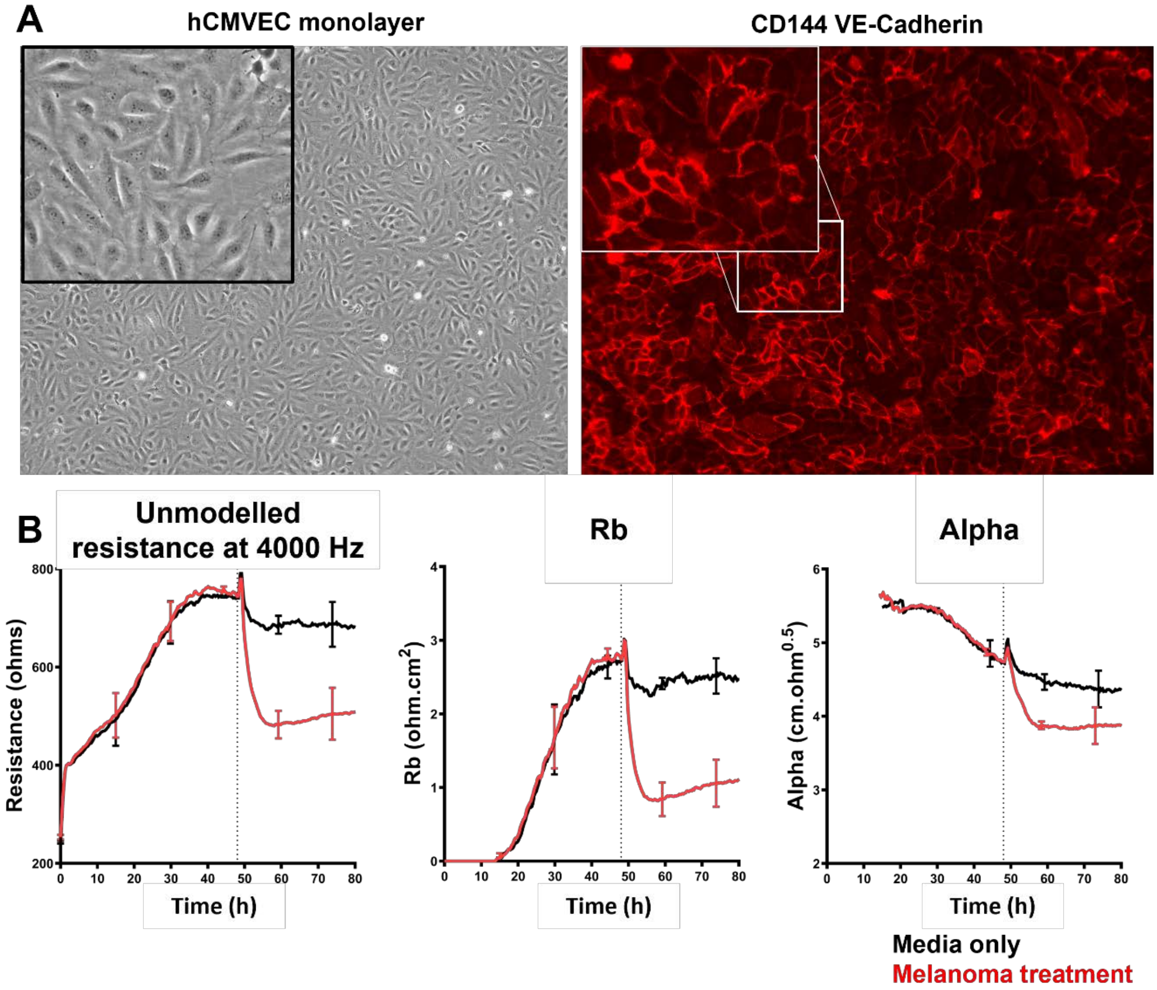

Figure S1. Representation of hCMVECs, the barrier forming brain endothelial cell line. (A)-Left shows the hCMVECs during cell culture, representing their confluent and typical monolayer formation. (A)-Right show hCMVECs fixed with 4% formalin at 48 hours post-seeding 20,000 cell per well in a 96 well plate, as per treatment assays conducted in this paper. These were stained for adherens junctional protein VE-Cadherin (CD144; #sc-9989, Santa Cruz, Dallas, Texas, USA) showing that stable junctions are formed. (B) Growth phase of the hCMVECs on ECIS representing that the resistance plateaus at approximately 48 hours, once the junctions are formed. The Rb or paracellular barrier begins forming after 10 hours as it takes time for the hCMVECs to become confluent first and then form junctions between each other. The Rb also stabilizes at around 48 hours. Alpha or basolateral adhesion also begin to form post 10 hours.

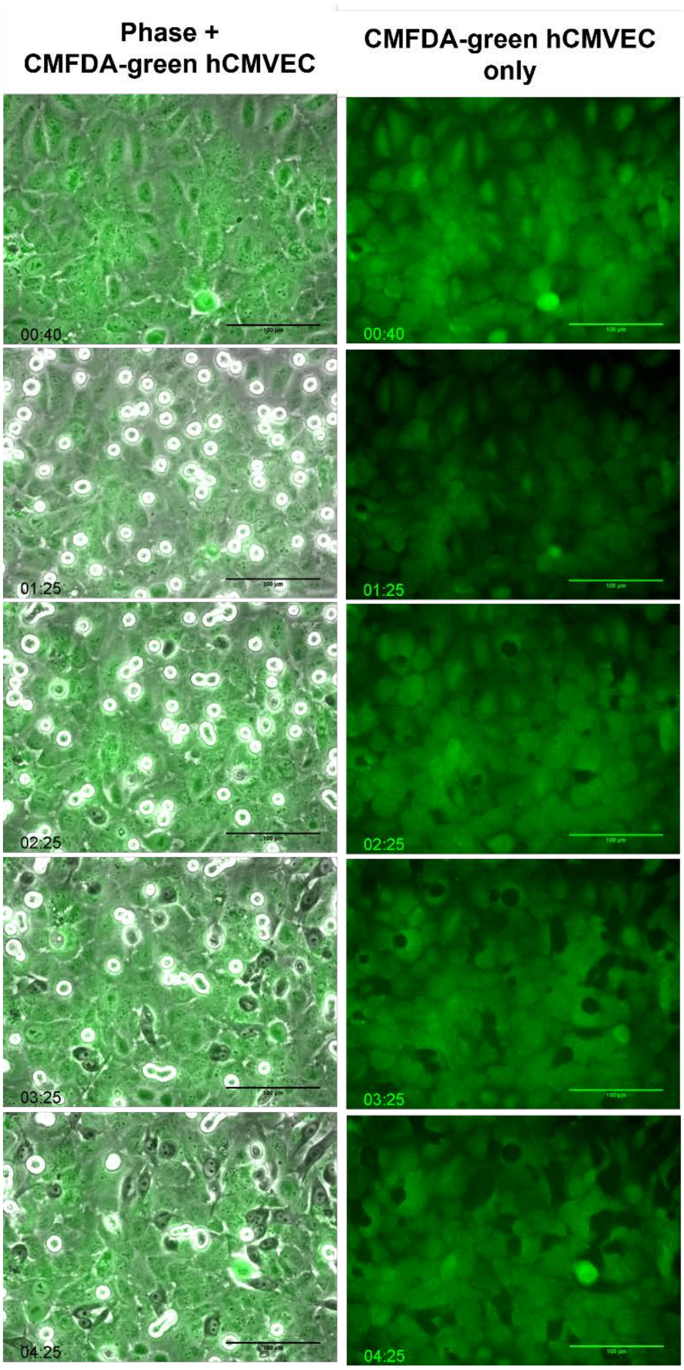

Figure S2. Time-lapse series illustrating melanoma integration into the brain endothelial monolayer. Left panel shows the endothelial cells, hCMVECs, stained with CMFDA-green (5-chloromethylfluorescein diacetate). These are merged with phase images to visualize unstained melanoma cells. Right panel shows the same but with CMFDA-green only to showcase just the endothelial cells. Spherical melanoma cells are added after 1 hour, after which holes and gaps start to appear within the endothelial monolayer. Each gap in the right panel can be traced back to a melanoma cell in the left panel. The time lapse of this is published in [5].

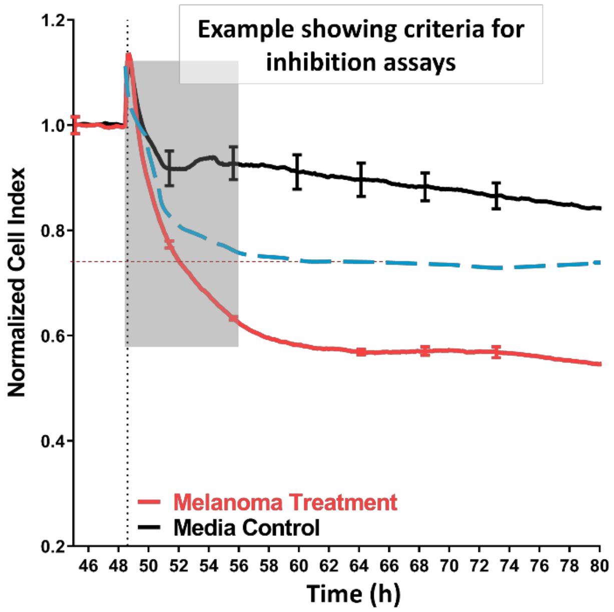

Figure S3. Example showing the criteria or cut-off for an expected, advantageous effect of inhibiting disruptive proteases. If a protease of interest were majorly involved, we would expect to see an improvement in brain endothelial Cell Index or resistance, as depicted by the blue dashed line. Note this effect is of a lesser magnitude and observed during the initial period of melanoma treatment. The effect starts within the initial period of insult caused by the melanoma cells (within the grey box) and is reduced by approximately 25% of the normalized start point 1 (horizontal red line). Therefore, if a protease were involved in the initial disruption caused by the melanoma cells, we would expect to see an effect as indicated by the blue curve especially in the initial hours (grey box).

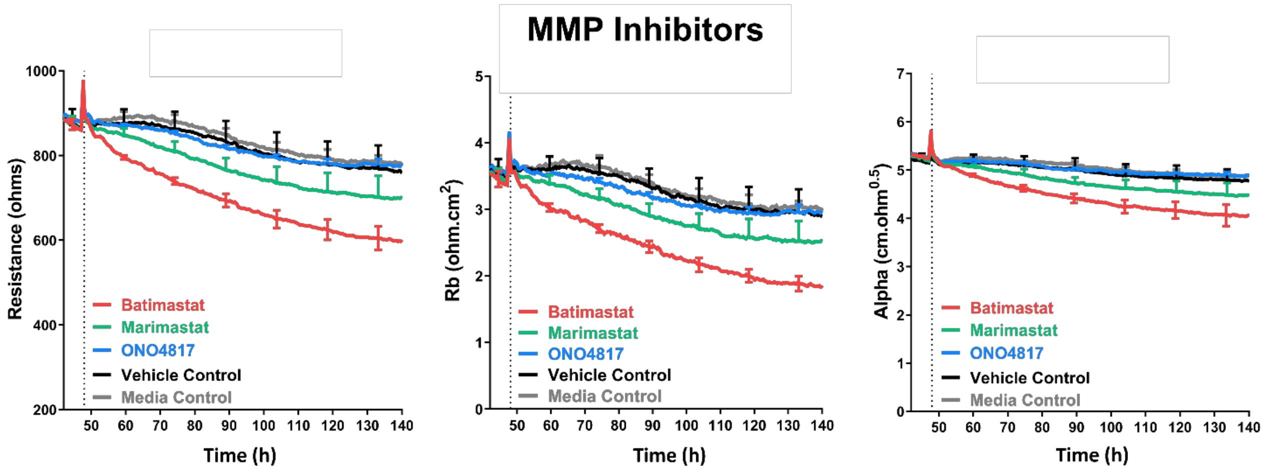

Figure S4. Effect of MMP inhibitors on the brain endothelial cells as measured by ECIS. Rb = paracellular resistance, Alpha = basolateral resistance.

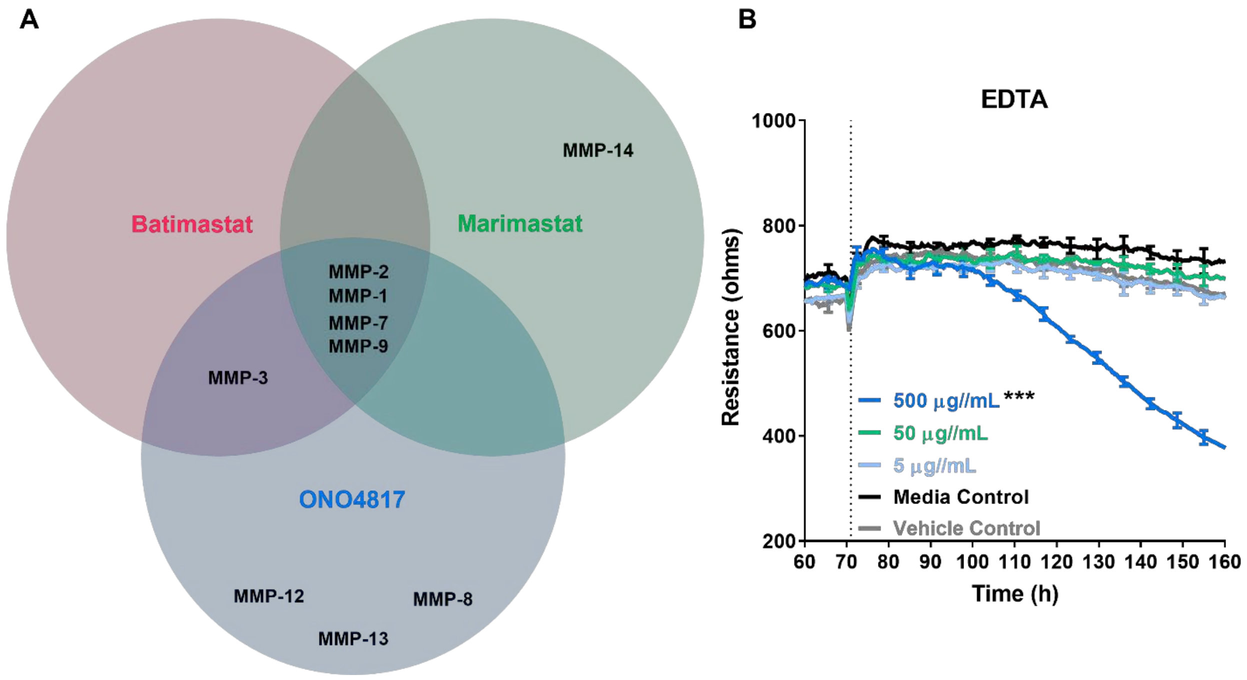

Figure S5. (A) Overlapping blocking specificity of MMP inhibitors with low nM potency. (B) Toxicity of EDTA after 120 hours in culture.

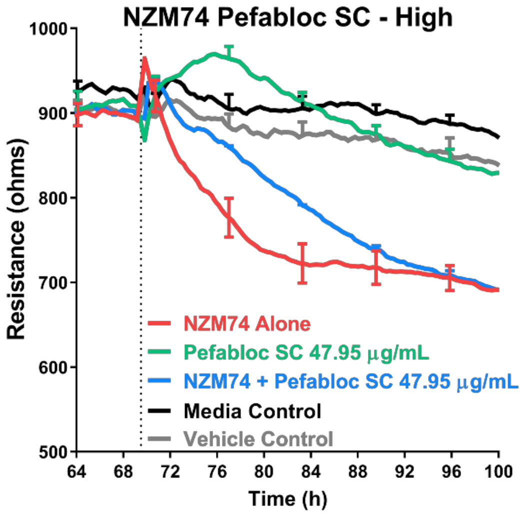

Figure S6. Effect of Pefabloc SC at a higher concentration of 49.75 µg/ml on NZM74 mediated barrier disruption.
